# Effects of Local and Landscape Factors on Population Dynamics of a Cotton Pest

**DOI:** 10.1371/journal.pone.0039862

**Published:** 2012-06-29

**Authors:** Yves Carrière, Peter B. Goodell, Christa Ellers-Kirk, Guillaume Larocque, Pierre Dutilleul, Steven E. Naranjo, Peter C. Ellsworth

**Affiliations:** 1 Department of Entomology, The University of Arizona, Tucson, Arizona, United States of America; 2 Kearney Agricultural Center, University of California Cooperative Extension, Parlier, California, United States of America; 3 Department of Plant Science, McGill University, Sainte-Anne-de-Bellevue, Quebec, Canada; 4 Arid-Land Agricultural Research Center, USDA-ARS, Maricopa, Arizona, United States of America; University College London, United Kingdom

## Abstract

**Background:**

Many polyphagous pests sequentially use crops and uncultivated habitats in landscapes dominated by annual crops. As these habitats may contribute in increasing or decreasing pest density in fields of a specific crop, understanding the scale and temporal variability of source and sink effects is critical for managing landscapes to enhance pest control.

**Methodology/Principal Findings:**

We evaluated how local and landscape characteristics affect population density of the western tarnished plant bug, *Lygus hesperus* (Knight), in cotton fields of the San Joaquin Valley in California. During two periods covering the main window of cotton vulnerability to *Lygus* attack over three years, we examined the associations between abundance of six common *Lygus* crops, uncultivated habitats and *Lygus* population density in these cotton fields. We also investigated impacts of insecticide applications in cotton fields and cotton flowering date. Consistent associations observed across periods and years involved abundances of cotton and uncultivated habitats that were negatively associated with *Lygus* density, and abundance of seed alfalfa and cotton flowering date that were positively associated with *Lygus* density. Safflower and forage alfalfa had variable effects, possibly reflecting among-year variation in crop management practices, and tomato, sugar beet and insecticide applications were rarely associated with *Lygus* density. Using data from the first two years, a multiple regression model including the four consistent factors successfully predicted *Lygus* density across cotton fields in the last year of the study.

**Conclusions/Significance:**

Our results show that the approach developed here is appropriate to characterize and test the source and sink effects of various habitats on pest dynamics and improve the design of landscape-level pest management strategies.

## Introduction

Landscape transformation resulting from increases in the extent and intensity of agricultural activities is often associated with greater pest pressure and use of environmentally-disruptive pesticides [1]. As increased demand for food will continue to favor agricultural intensification for decades, the vulnerability of intensively managed agro-ecosystems may increase in the future [2]. Accordingly, the need for sustainable pest management is increasing interest in manipulating agricultural landscapes to disrupt the capacity of pests to infest crops [3]–[5]. Much work has been done at the field scale to understand how spatial arrangements of vegetation affect pest movement and population dynamics [6], [7]. However, much less information is available on effects of landscape heterogeneity on pest metapopulation dynamics [8], [9].

The demographic impact on population density of particular habitats for other patches in the landscape has been characterized based on the local balance between birth and death rates and immigration and emigration [10], [11]. Here, we define source habitats as areas that increase pest density in fields of a specific crop, while sink habitats are areas that reduce pest density in fields of that crop. Many significant polyphagous pests exploit a wide array of crops and uncultivated habitats that may act as sources or sinks for focal crops at some time during the growing season [12].

*Lygus* spp. (Hemiptera: Miridae) provide classical examples of source-sink dynamics resulting in crop damage through spatial subsidies [13], [14]. After overwintering in uncultivated habitats with weedy host plants, adults colonize crops such as alfalfa (*Medicago sativa* L.) and safflower (*Carthamus tinctorius* L.), where large populations develop in the spring and early summer. Adults move to cotton when alfalfa is harvested or safflower matures and becomes less suitable [13], [14]. Insecticides are typically applied in cotton following such dispersal because cotton is highly vulnerable to *Lygus* feeding during fruit formation. Landscape-based management to reduce *Lygus* populations in cotton has included lessening the source potential of certain crops and uncultivated habitats, or planting alternative hosts in cotton fields to divert *Lygus* feeding from cotton [15], [16]. Another management practice could involve manipulating the spatial arrangements of source and sink habitats [16], [17]. However, the source or sink potential of habitat patches can vary dramatically in space and time, and the consequences of this variation on the spatial structure of pest populations remain largely unknown [12], [18].

The goals of this study were to characterize temporal variation in effects of local and landscape characteristics on the population density of *L. hesperus* in cotton, and to assess whether the spatial pattern of *L. hesperus* populations can be predicted despite this temporal variation in the San Joaquin Valley of California. The main period of cotton vulnerability to *L. hesperus* attack occurs between June and August. The source and sink characteristics of particular habitats may vary during this period, due to changes in suitability of host plants or harvest. Among-year variation in abundance of crops could also influence the source and sink characteristics of habitats if habitat choice of migrants is affected by the relative availability of habitats. We thus used geographic information system (GIS) technology combined with spatial statistics to evaluate: 1) within- and among-year variations in effects of cotton field characteristics and of certain crops and uncultivated habitats, 2) the spatial scale of the associations between abundance of the habitats and *L. hesperus* density in cotton, and 3) how the local and landscape factors combine to determine *L. hesperus* population density in cotton.

## Methods

### Ethics Statement

No specific permits were required for the described field studies.

### Field Sites and GIS Mapping of Agricultural Fields

In 2007, 2008, and 2009, we sampled *L. hesperus* in cotton fields once a week between June and August (Table 1), which is the main period of cotton vulnerability to *L. hesperus* attack. Most fields were sampled in only one year (*n*  = 128), although two fields sampled in 2007 were resampled in 2008 and two other fields sampled in 2007 were sampled again in 2009. These cotton fields were located in the Fresno and Kings Counties of the San Joaquin Valley (see Fig. S1). The study area was larger in 2007 than in subsequent years because cotton fields were more extensively distributed in 2007 than in 2008 and 2009. Location and shape of agricultural fields were determined with U.S. Department of Agriculture’s Farm Service Agency common land unit maps [19] and validated from the ground with a Global Positioning System (GPS) at a resolution of 5 m. Over the three years, the average shortest distance between pairs of sampled cotton fields and area of sampled cotton fields varied between 2.5 and 3.9 km and 62.8 and 91.4 ha, respectively (Table 1). Pima cotton (*Gossypium barbadense* L.) was more frequently planted than Upland cotton (*G. hirsutum*) in the study area.

**Table 1:** Characteristics of cotton fields sampled for western tarnished plant bug, *Lygus hesperus.*

Year*	FieldArea†	Closestdistance†	Firstperiod‡	Secondperiod‡	Floweringdate¶	% Pimacotton	Insecticidesprays†	*Lygus*density† §
2007	67.8 (7.1)	3.9 (0.3)	10 Jun (6)	22 Jul (5)	30 Jun	85	1.2 (0.10)	4.3 (0.5)
2008	62.8 (4.5)	2.7 (0.2)	15 Jun (5)	20 Jul (4)	10 Jul	92	7.8 (0.4)	13.3 (1.4)
2009	91.4 (6.6)	2.5 (0.1)	9 Jun (5)	14 Jul (5)	23 Jun	89	3.9 (0.4)	5.3 (0.4)

Variables shown are average field area (ha), average closest distance between pairs of sampled cotton fields (km), date of initiation of first and second sampling periods, average date of initiation of flowering, percentage of sampled fields planted to Pima cotton, average number of insecticide sprays and average *Lygus* density (calculated per 100 sweeps to facilitate comparison with thresholds) for combined sampling periods in each year.

* Number of fields sampled: 41 in 2007; 39 in 2008; 56 in 2009.

† Standard error in parentheses.

‡ Date is for onset of sampling period; number of weeks sampled per period is in parentheses.

¶ Range associated with average flowering date was 22 Jun–7 Jul in 2007, 7 Jul–12 Jul in 2008, and 17 Jun–2 Jul in 2009.

§ Suggested thresholds for *Lygus* spraying depend on cotton phenology [48]. Number of individuals per 100 sweeps that would trigger spraying is: >4–8 adults (early squaring); >14–20 individuals with at least two nymphs (bloom); and >20 individuals with nymphs present (boll filling).

Winters in the San Joaquin Valley are moist and foggy but summers are hot and dry. Non-reproductive *L. hesperus* adults overwinter in uncultivated habitats and move into crops in late winter and spring when uncultivated vegetation starts to dry up [20]. Here we focused on the source and sink potential of uncultivated habitats and crops known to harbor significant *L. hesperus* populations in the study area [21]: cotton, forage alfalfa, safflower, seed alfalfa, sugar beet (*Beta vulgaris* L.), and tomato (*Solanum lycopersicum* L.) (see Fig. S2). Crops in fields <3 km from the edge of sampled cotton fields were identified by visual survey from the ground. Uncultivated habitats within this distance were identified from geographically referenced data (see below).

### Within-fields Variables

*Lygus* spp. prefer to feed on developing cotton flower buds and young fruits [22]. We thus investigated the impact of cotton flowering date in addition to effects of landscape composition. Date of initiation of flowering was determined with planting dates obtained from producers and a model based on accumulation of degree-days [23]. We also evaluated effects of insecticide sprays applied during the sampling periods. Insecticide data were provided by cotton producers.

### Sampling Method

Sampled cotton fields were divided in four quadrants with each quadrant sampled weekly. Samples were collected starting at least 25 m inside each quadrant and consisted of 100 sweeps. The upper part of plants was sampled because it is a preferred feeding and oviposition area for *L. hesperus* [24]. The number of adults from the 400 sweeps was recorded for each week and field.

### Landscape Analysis

Fields were mapped using ArcGIS version 10.0 [25]. Roads and urban areas were overlaid on field maps. We drew twelve concentric rings around the edge of each sampled cotton field. The first ring had a distance from the field edge of 250 m and the distance of each subsequent ring increased by 250 m; the largest ring had a distance of 3000 m. The area of each crop type (m^2^) between the edge of a sampled cotton field and a ring was calculated with ArcGIS. Uncultivated vegetation within rings was primarily found along irrigation canals and roads, in riparian areas, near urban developments, or in rangelands. Inspection from the ground and with high-resolution imagery in Google Earth [26] showed that such uncultivated habitats mainly comprised grass or weeds and shrubs, and thus plausibly contained *L. hesperus* hosts. Area of uncultivated habitats in each ring was calculated by subtracting the area occupied by agricultural fields, roads and urban development from the area of the ring.

### Data Analysis

#### Source and sink effects

Here we use the slope of the statistical association between *L. hesperus* density in sampled cotton fields and the area of a habitat type surrounding the cotton fields to infer source or sink effects, whereby a significant negative association indicates a sink effect and a positive association a source effect. The source and sink potential of particular habitats could vary during the cotton vulnerability period (i.e., June to August), due to changes in host suitability or harvest. To evaluate potential variation in the associations between areas of habitat types and *L. hesperus* density during this period, the mean number of *L. hesperus* adults sampled per week in each field was averaged over two successive periods. Duration of the first and second periods varied between four and six weeks depending on year (Table 1). *L. hesperus* density did not differ significantly between Pima and Upland cotton in any period (2-sample *t*-tests, *P-*values >0.1). Furthermore, preliminary analyses conducted at all scales (method described below) indicated qualitatively similar effects of Pima and Upland cotton. The two species of cotton were thus considered as a single crop for analysis.

The number of potential explanatory variables (i.e., nine: areas of six crops and uncultivated habitats, cotton flowering date, and number of insecticide applications) was relatively high, compared to the number of experimental units (Table 1, between 39 and 56 fields sampled per year). Therefore, we first used stepwise regression (with forward selection and backward elimination) to select a subset of relevant explanatory variables. Average *L. hesperus* density in a field was the response variable, and area of each crop, area of uncultivated habitats, cotton flowering date (number of days since January 1 of each year), and number of insecticide sprays in cotton were the candidate explanatory variables. For each period of the three years, we performed an analysis at each of the 12 spatial scales (ring distance from 250 to 3000 m). Variables with significant explanatory effect (*P*<0.05) at one or more scales were retained for subsequent analysis.

Multiple regression was then used to evaluate the association between *L. hesperus* density and the explanatory variables selected in the stepwise procedure. For a given period and year, the same multiple regression model was fit for all 12 scales. Partial F-tests were used to assess significance of explanatory variables included in the model. As in Carrière et al. [17], [27], we used rank-based statistics in stepwise and multiple regression analyses because assumptions of normality and homogeneity of variance were not met by the raw data. Statistical analysis was adjusted for spatial autocorrelation when required (see below).

#### Scale of source and sink effects

A significant association between area of a habitat type and *L. hesperus* density is expected if the area in a ring comprises patches that affect *L. hesperus* density in sampled cotton fields, but statistical significance is expected to decline once the scale of analysis exceeds the distance at which patches affect *L. hesperus* density [28]. Therefore, we used the largest ring at which a significant effect was found for a habitat type to infer the scale of source or sink effects. Because larger rings included patches present in smaller rings, this procedure may overestimate the scale of source and sink effects through “carry-over effects”. To assess this possibility, we performed additional multiple regression analyses with two adjacent rings of increasing width (from 250 m to 1500 m). Pairs of adjacent rings do not share patches because the larger rings do not include patches in the smaller rings (e.g., a 250 m-wide ring includes patches from edge of field up to a distance of 250 m, while a 500 m-wide ring includes patches at distance between 250 m and 500 m), so the maximum scale at which a significant association is observed in two-ring analyses is not affected by carry-over effects [17], [27]. Nevertheless, two-ring analyses may have lower statistical power than single-ring analyses because the number of explanatory variables required to investigate source and sink effects in the former is doubled (e.g., with six crops, twelve explanatory variables are used in multiple regression, instead of six). As expected, two-ring analyses detected a lower number of significant effects than single-ring analyses. However, across periods and years, the scale of effects detected in both single-ring and two-ring analyses did not differ significantly (paired *t*-test, *P*  = 0.33), indicating that carry-over effects were not important. Here we only report results from single-ring analyses across the 12 scales. Among-habitat differences in average scale of source and sink effects (across periods and years) were assessed with one-way ANOVA [29].

#### Prediction of L. hesperus density

The goal of the predictive model was to show that factors with consistent effects were sufficient to predict spatial variation in *L. hesperus* density, even when other important factors were not considered in the predictive model (see **Discussion**). Analyses of data from the first two years revealed that the areas of cotton, uncultivated habitats and seed alfalfa, and date of flower initiation had consistent effects on *L. hesperus* density (see **Results**). Safflower also had consistent effects during the first two years, but its effects changed in the third year. Therefore, only the first four variables were included in a predictive model derived from data obtained in the first two years (see **Discussion**).

Before pooling data from the first two years to formulate the predictive model, one-way ANOVAs with year as the classification factor were performed to remove between-year variations in the response and explanatory variables. Standardized residuals from these ANOVAs (i.e., centered data divided by the standard deviation within each year) provided the response and explanatory variables in rank-based multiple regression analyses, which evaluated the association between *L. hesperus* density (calculated over the main period of cotton vulnerability between June and August) and areas of the cultivated and uncultivated habitats and flowering date at each of the 12 scales. Thus, data from 2007–2008 were used to analyze the association between among-site variations in *L. hesperus* density and among-site variations in areas of habitat types near each sampled field and date of cotton flower initiation.

The regression model with the highest coefficient of determination (*R^2^*) was selected for prediction of *L. hesperus* density in 2009. Values of the explanatory variables at the corresponding scale for each sampled field in 2009 were substituted in the multiple regression model to calculate predicted values of ranks for *L. hesperus* density. A rank-based simple linear regression was then used to assess the association between predicted and observed values of *L. hesperus* density in 2009.

#### Spatial autocorrelation

In each stepwise and multiple regression analysis, and in the analysis of predicted versus observed *L. hesperus* density, semivariograms were computed to quantify and analyze spatial autocorrelation in *L. hesperus* density and other variables across fields [30]. By assessing spatial patterns in residuals (response variable) and partial residuals (explanatory variables), we evaluated spatial autocorrelation at all scales and corrected for potential non-independence of observations. Spatial autocorrelation was accounted for in tests of significance through the use of effective sample sizes, in modified *t*- and *F*-tests performed in simple linear correlation analysis, stepwise regression, and multiple regression [30]–[32]. Programs for these statistical analyses were written in Matlab [33].

## Results

Composition of the landscape in rings surrounding the sampled cotton fields varied during the three years. The main changes involved a decrease in the area occupied by cotton and uncultivated habitats from 2007 to 2008 and 2009, and an increase in the area occupied by safflower and tomato in 2008 compared with 2007 and 2009 (Table S1). Other differences included greater *L. hesperus* population density and use of insecticides, and later cotton flowering dates in 2008 than in 2007 and 2009 (Table 1). Abundance of cotton, seed alfalfa, and uncultivated habitats were frequently and consistently associated with *L. hesperus* density in sampled cotton fields across sampling periods and years (Table 2). The significant negative associations for cotton and uncultivated habitats indicate that these habitats were sinks for *L. hesperus*. On the other hand, the significant positive associations for seed alfalfa indicate that this crop was a source of *L. hesperus* for cotton fields.

**Table 2:** Average regression coefficient for the association between *Lygus* density in sampled cotton fields and abundance of crops and uncultivated habitats, estimated for two sampling periods in three years.

Habitat	First period			Second period		
	2007*	2008	2009	2007	2008	2009
Cotton	−0.39 (0.03, 11)	−0.27 (NA†, 1)	−0.38 (0.01, 9)	−0.40 (0.03, 11)	−0.41 (0, 2)	−0.46 (0.01, 12)
Forage alfalfa	0.31 (0.005, 5)	−0.4 (NA, 1)				
Uncultivated habitats	−0.21(0.02, 5)			−0.45 (0.05, 4)		−0.31 (0.06, 2)
Safflower	0.14 (NA, 1)	0.34 (0.02, 9)	−0.34 (0.006, 5)			−0.31 (0.005, 2)
Seed alfalfa	0.47 (0.06, 8)	NA†		0.74 (0.02, 12)	NA	0.59 (0.06, 7)
Sugar beet		NA	NA	0.12 (0.13, 4)	NA	NA
Tomato	−0.22 (0.03, 3)					
Flowering date		0.44 (0.01, 12)		0.25 (0, 3)		
Insecticide sprays						−0.34 (0.01, 8)

Effects of flowering date and insecticide sprays are also shown.

* After correcting for spatial autocorrelation, criterion for assessing significance of regression coefficients was P<0.1. Number reported is average of the significant regression coefficients in analyses performed at the 12 scales. Parentheses contain standard error followed by number of significant regression coefficients.

† NA: Standard error was not calculated because a single coefficient was significant, or a crop was not included in analyses because it was rare in rings.

Abundance of safflower was frequently associated with *L. hesperus* density in cotton fields. However, the significant coefficients were positive in 2007 and 2008 and negative in 2009, indicating that this crop was a source of *L. hesperus* for cotton in the first two years but a sink in the last year. Forage alfalfa also had variable effects, as the significant coefficients were positive in 2007 and negative in 2008. Areas of sugar beet and tomato were associated with *L. hesperus* density only once, although sugar beet was only included in analyses in 2007 because it was rare in other years (Table 2).

**Figure 1:**
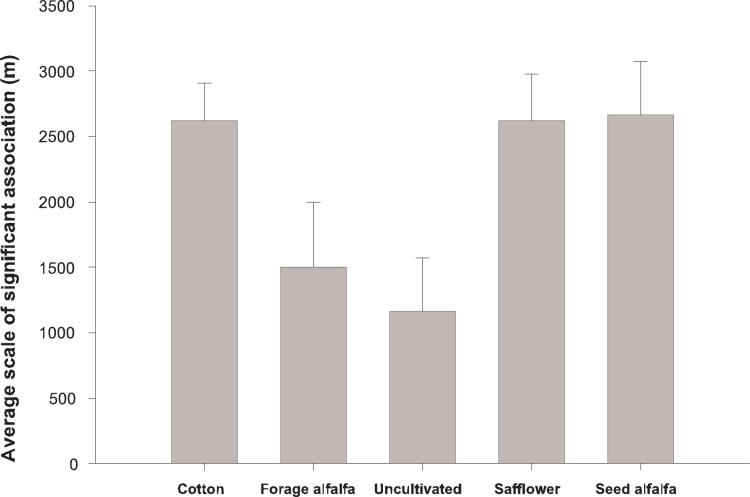
Scale of association between *Lygus* density in cotton fields and abundance of surrounding habitats. Average scale (mean + SE) for habitats found to have significant effects in a least two of the six analyses are shown. Standard errors were derived from the ANOVA.

The scale at which abundances of cultivated and uncultivated habitats were significantly associated with *L. hesperus* density differed among habitats (*F*  = 3.35, *d.f.*  = 4, 13, *P* = 0.043). Cotton, safflower and seed alfalfa affected *L. hesperus* density over larger spatial scales than forage alfalfa and uncultivated habitats (Fig. 1). The associations between date of flower initiation and *L. hesperus* density were positive and significant in 2007 and 2008 (Table 2). The number of insecticide sprays was negatively associated with *L. hesperus* density only once, in 2009.

**Figure 2:**
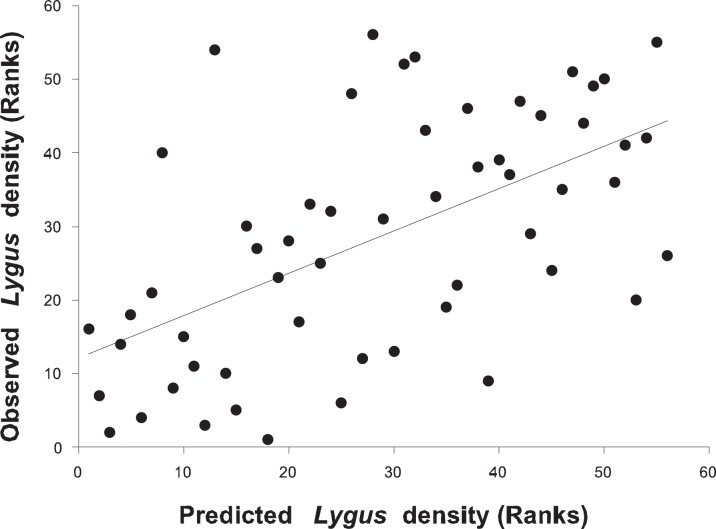
Association between observed and predicted density of *L. hesperus* in cotton fields. Rank-based regression analysis was used to evaluate the association across 56 cotton fields sampled in 2009. The model used to calculate predicted values of ranks for *Lygus* density was: *Lygus* density = 44.3–0.41 (area of cotton) +0.096 (flowering date) +0.25 (area of seed alfalfa) –0.073 (area of uncultivated habitats).

The coefficient of determination of the multiple regression model including the abundances of cotton, seed alfalfa, uncultivated habitats and flowering date varied from 16.7 to 24.4% across the 12 scales. The *R^2^* value was highest at the 2750-m scale, which was thus the scale used to test the predictive model. For fields sampled in 2009, the association between predicted and observed ranks of *L. hesperus* density was positive and significant (Fig. 2, *R* ^2^ = 33.2%, *F* = 26.9, *d.f.* = 1, 54, *P*<0.0001, spatial autocorrelation was not significant in this analysis).

## Discussion

Landscapes dominated by annual crops represent networks of ephemeral patches for multivoltine generalist pests that track the availability and suitability of resources during the growing season. Although control of generalist pests has typically hinged on within-field management, migration among patches often affects pest population dynamics locally and regionally [8], [12], [34]. Here we found that the abundance and distribution of cotton, uncultivated habitats and seed alfalfa surrounding the monitored cotton fields, and date of cotton flower initiation had consistent within- and among-year effects on density of *L. hesperus*. These factors were sufficient to predict *L. hesperus* density across cotton fields in the Fresno and Kings Counties in the San Joaquin Valley of California in 2009. Flowering date of cotton may have affected *L. hesperus* density because it synchronized dispersal of individuals with presence of the most suitable cotton phenological stages [12]. Thus, each factor included in the predictive model probably influenced among-patch migration, suggesting that a landscape-based approach will be useful to manage *L. hesperus* populations in cotton.

The consistent negative association between abundance of cotton and *L. hesperus* density in sampled cotton fields may be explained by the low density of *L. hesperus* in cotton compared to other habitats [17], [21], [35] and the low attractiveness of cotton compared to other hosts [14], [35], [36]. Control of *L. hesperus* populations with insecticides during the fruiting period (Table 1) could account at least in part for the low *L. hesperus* densities in cotton. Conversely, the consistent positive association between abundance of seed alfalfa and *L. hesperus* density in cotton likely occurred because seed alfalfa is an attractive and suitable *L. hesperus* host and many individuals disperse from this crop when irrigation is terminated before harvest. Similar sink and source effects of cotton and seed alfalfa were respectively found in a one-year study conducted in an arid agricultural landscape of Central Arizona [17].

A negative association between abundance of uncultivated habitats and *L. hesperus* density occurred in both parts of the cotton vulnerability period. The reasons for this pattern are not clear. Most uncultivated habitats harbor sparse *L. hesperus* populations in years with low rainfall, suggesting that uncultivated hosts should not be significant sources during these years [16]. However, large *L. hesperus* populations can develop in uncultivated habitats in years with high rainfall [37]. In such years, uncultivated habitats may attract and retain *L. hesperus* until July because high moisture availability postpones weed dry-up and *L. hesperus* prefers some weeds over cotton [35], [37]. Yet, uncultivated habitats should become sources when weeds eventually dry up at the end of July. Accordingly, it seems that seasonal changes in suitability and attractiveness of uncultivated hosts may not account for the negative associations of *L. hesperus* with uncultivated habitats found in the second sampling period.

Our finding that abundances of forage alfalfa and safflower were frequently associated with *L. hesperus* density in cotton indicates that a better understanding of landscape effects of management practices in these crops could greatly contribute in managing *L. hesperus* populations in cotton. Forage alfalfa and safflower have been managed to reduce *L. hesperus* movement to cotton since the mid 1960s in the San Joaquin Valley [13], [21]. To reduce movement from forage alfalfa to cotton, strips of alfalfa are left at harvest to retain adults in the uncut portions of fields [38]. Insecticides can also be applied to safflower before harvest to limit adult emigration [39]. Although these practices do not significantly improve yield or quality of the treated crop, they increase insecticide use and complexity of crop management. Consequently, they are only profitable for producers that also grow cotton, or when cotton producers compensate alfalfa and safflower producers for extra costs and difficulties associated with *L. hesperus* management. On average, about 50% of producers manage alfalfa and safflower to reduce *L. hesperus* migration to other crops in the San Joaquin Valley, although use of these management practices varies across years and counties [16], [40], [41]. Accordingly, the inconsistent source and sink effects of forage alfalfa and safflower observed here may have been due to spatial and temporal variations in implementation of practices to reduce the source effect of these crops. Indeed, there is evidence that many safflower fields were treated with insecticides before harvest in 2009 but few in 2007 and 2008 [41]. This may explain why safflower was a source in 2007 and 2008 but a sink in 2009.

The influence of other ecological factors such as natural enemy induced mortality on these observed landscape patterns is unclear. Although many species of parasitoids and generalist predators attack *Lygus* spp. in a variety of habitats in the U.S. [42], [43], the impact of these natural enemies on pest dynamics is not well understood [42]. Recent work shows that abundance of *Geocoris* spp. in cotton is associated with reductions in immature stages of *L. hesperus* [44]. Species of parasitoids from Europe have become established in limited areas of central California [45], but their impacts appear restricted to strawberry production in coastal regions. Ongoing work is examining the influence of landscape factors on the dynamics of natural enemies. This work may allow us to better explain the spatial patterns observed here, including the sink effects of uncultivated habitats on *L. hesperus* populations in cotton.

Number of insecticide applications in cotton was rarely associated with *L. hesperus* density across cotton fields. Birth and immigration contribute to population growth in a patch while death and emigration reduce it [10], [11]. Insecticides are generally applied when *L. hesperus* density exceeds a specific threshold (Table 1). If immigration rates varied among cotton fields and fields with high *L. hesperus* influx received more insecticides, immigration and mortality from insecticides may have often compensated each other. Thus, insecticides may contribute in reducing *L. hesperus* damage to cotton, especially by sedentary nymphs, but not in reducing populations of the mobile adult stage over time, as observed here in most sampling periods and years.

Independent sets of data were used to select factors included in the predictive model (i.e., data from 2007–2008) and evaluate accuracy of this model (i.e., data from 2009). However, safflower was excluded from the predictive model because analyses revealed that effects of this crop on *L. hesperus* density changed in 2009. The change in effects of safflower in 2009 was likely due to changes in safflower management in that year (see above). Thus, in the strictest sense, formulation and evaluation of the predictive model were not accomplished with independent data. Nevertheless, for the four factors included in the predictive model, an independent set of data was used to evaluate the quality of model predictions, in accord with recommended practices for development of predictive distribution models [46]. Importantly, excluding safflower from the predictive model here is appropriate because the goal of this model was to show that the four factors with consistent effects were sufficient to predict spatial variation in *L. hesperus* density, even when other important factors such as the abundance of safflower and forage alfalfa were not considered. The statistical approach used here was recently applied to predict spatial variation in the evolution of resistance to an insecticide in *Bemisia tabaci* [28]. Taken together, these studies indicate that such approach will be useful for the development of spatially-explicit integrated pest management.

The associations between abundances of particular habitats (e.g., forage alfalfa and safflower) and *L. hesperus* density in cotton varied among years. In the absence of knowledge on the cause of such temporal variation, manipulation of the spatial arrangement of these habitats is difficult. Thus, a fundamental question in the design and implementation of landscape-level pest management strategies is whether the modification of a limited number of factors with consistent effects will be sufficient to produce the desired outcome. A positive answer to this question is suggested when a statistical model including these factors provides accurate prediction of pest population dynamics [46], [47]. Specifically, our demonstration that the spatial structure of *L. hesperus* populations in cotton was predicted with a model built on one local and three landscape factors with consistent effects across years increases the credibility of a landscape-based approach to manage this pest in the San Joaquin Valley.

The findings of this study indicate that patches of cotton, uncultivated habitats and seed alfalfa affects *L. hesperus* population density in cotton. Because increased abundance of cotton was associated with lower *L. hesperus* density in sampled fields, clumping cotton fields could contribute in reducing *L. hesperus* populations in cotton. The maximum scale of the significant negative associations between abundance of uncultivated habitats and *L. hesperus* density varied between 500 and 2000 m across periods and years. This indicates that groups of cotton fields at a distance <500 m from uncultivated habitats could harbor the lowest *L. hesperus* densities. Conversely, separating groups of cotton fields from seed alfalfa by more than 3 km (i.e., the maximum spatial scale of source effects of seed alfalfa observed here) could contribute in reducing *L. hesperus* populations in cotton. Cotton producers generally spread planting of fields over a few weeks. Because late flowering was associated with increased *L. hesperus* populations in cotton, fields located where *L. hesperus* immigration is expected to be high (e.g., near unmanaged safflower or seed alfalfa) could be planted earlier than fields in locations where *L. hesperus* dispersal is expected to be lower.

Pest infestations triggering applications of insecticides in specific crops often occur because polyphagous pests migrate from other source habitats [12]. Furthermore, the population dynamics of many polyphagous pests are likely affected by sink habitats, as these pests commonly prefer specific hosts or plants in particular phenological stages, and recurring application of insecticides in crops highly sensitive to damage and other management practices can drastically reduce their populations [12]. Accordingly, the metapopulation dynamics of many polyphagous pests depends on characteristics of the surrounding landscape and crop management practices applied to individual fields. Our results suggest that a systematic, spatially-explicit statistical approach taking into account the distribution of source and sink habitats and management practices in crop fields of interest can provide strong insights for designing landscape-level management strategies for such polyphagous pests.

## Supporting Information

Fig. S1 Cotton fields sampled for *Lygus hesperus* in the Fresno and Kings Counties of the San Joaquin Valley in 2007, 2008 and 2009. The insert shows location of the San Joaquin Valley in California (bottom left, dark area) and location of the study area in the San Joaquin Valley.
(TIF)

Fig. S2 Location of sampled cotton fields, crops assessed for source and sink effects, unidentified crops, uncultivated habitats, and urban areas in 2008. Rings with a distance from the field edge of 3000 m are shown. Across the three years, the largest uncultivated areas surrounding sampled cotton fields were rangelands (shown here in top-left ring), periphery of an airport (five center-right rings), and riparian zones (four lower-right rings).
(TIF)

Table S1 Mean % area occupied by crops assessed for source and sink effects, uncultivated habitats, and urban development in 3000-m rings surrounding sampled cotton fields. Standard errors are in parentheses.
(DOCX)
