# Supplementary figures and images for: Effects of Local and Landscape Factors on Population Dynamics of a Cotton Pest
Source: PLoS One. 2012 Jun 29;7(6):e39862. doi: 10.1371/journal.pone.0039862 (PMC3387197; doi:10.1371/journal.pone.0039862)

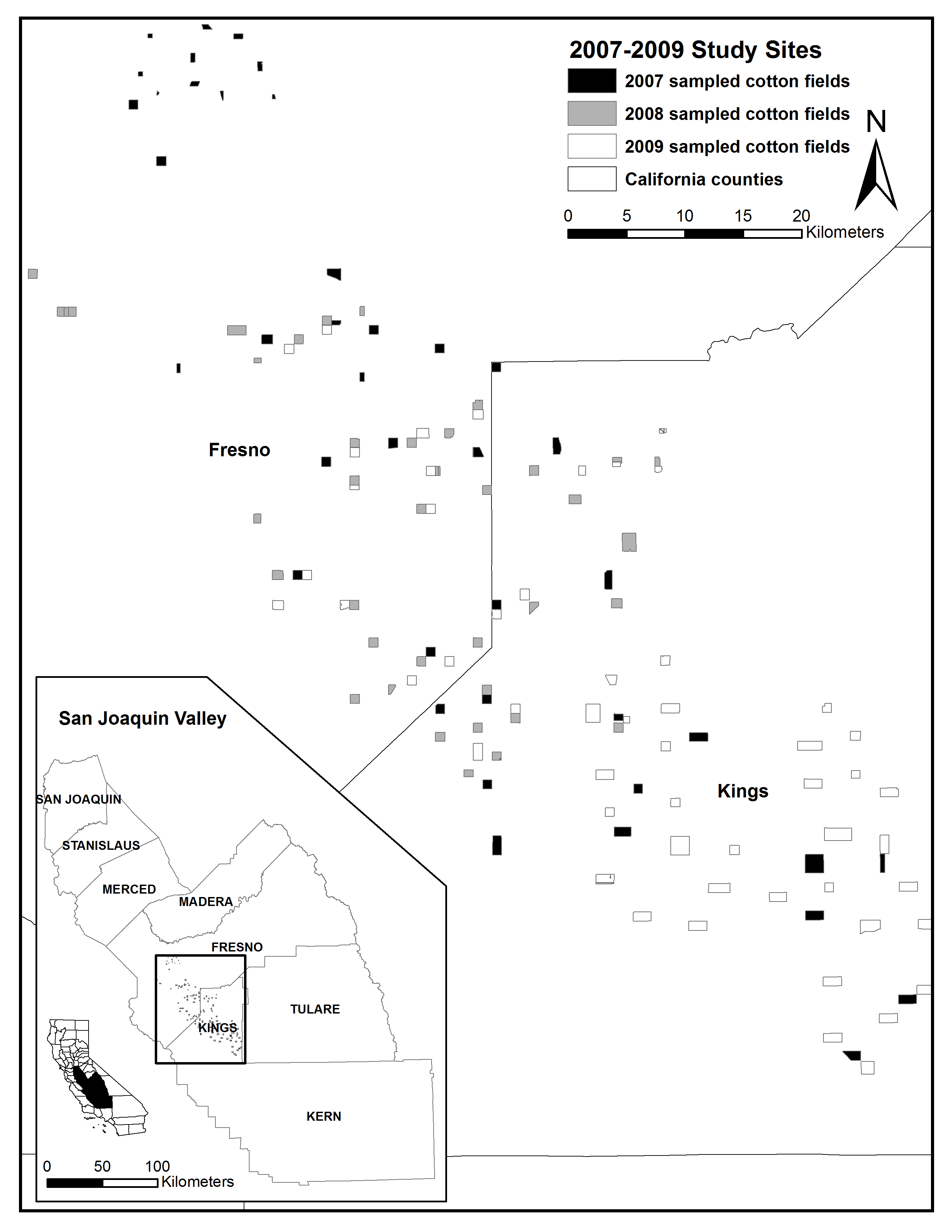

Supplement: Fig. S1 — Cotton fields sampled for Lygus hesperus in the Fresno and Kings Counties of the San Joaquin Valley in 2007, 2008 and 2009. The insert shows location of the San Joaquin Valley in California (bottom left, dark area) and location of the study area in the San Joaquin Valley. (TIF) [file pone.0039862.s001.tif]

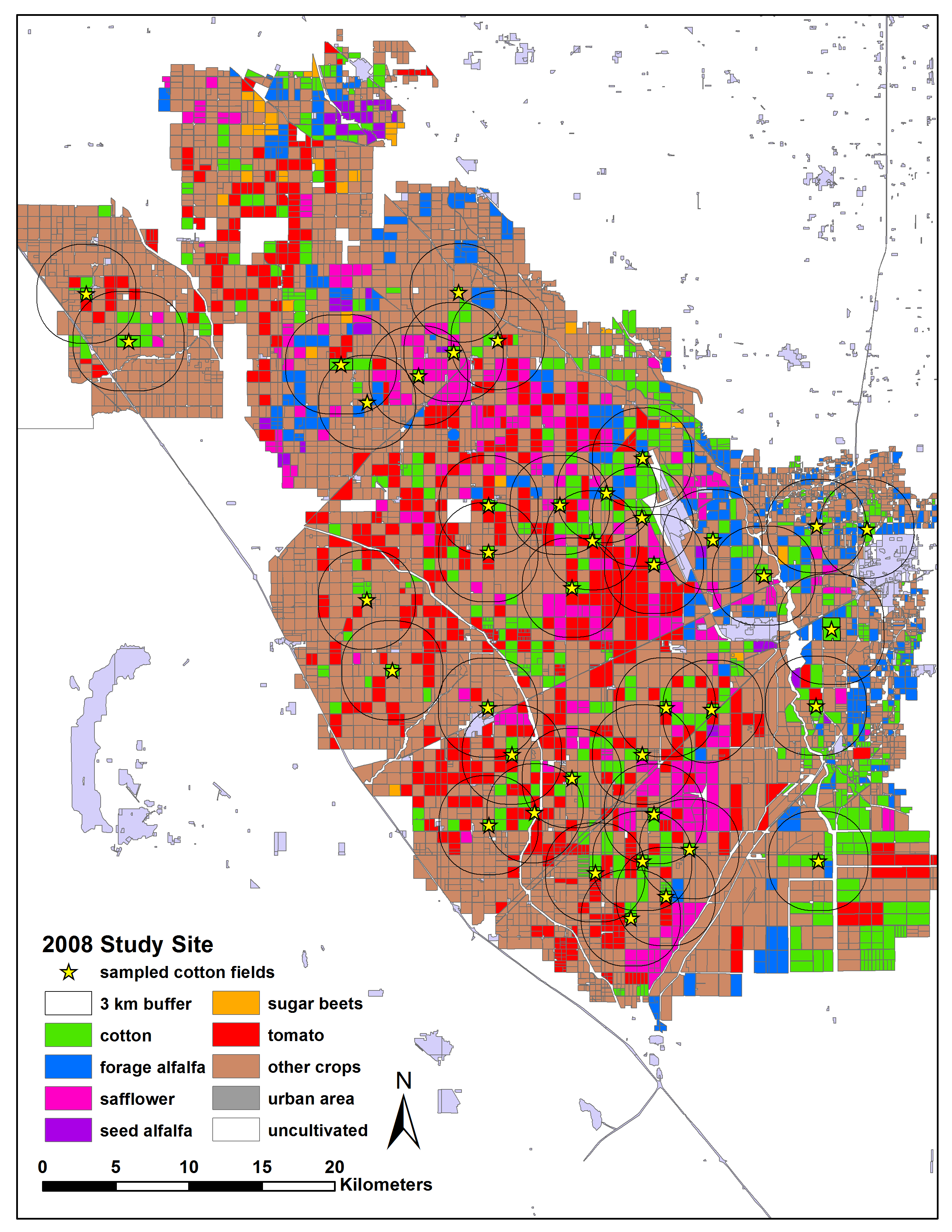

Supplement: Fig. S2 — Location of sampled cotton fields, crops assessed for source and sink effects, unidentified crops, uncultivated habitats, and urban areas in 2008. Rings with a distance from the field edge of 3000 m are shown. Across the three years, the largest uncultivated areas surrounding sampled cotton fields were rangelands (shown here in top-left ring), periphery of an airport (five center-right rings), and riparian zones (four lower-right rings). (TIF) [file pone.0039862.s002.tif]
