# Supplementary material for: Effects of Local and Landscape Factors on Population Dynamics of a Cotton Pest
Source: PLoS One. 2012 Jun 29;7(6):e39862. doi: 10.1371/journal.pone.0039862 (PMC3387197; doi:10.1371/journal.pone.0039862)
Supplement: Table S1 — Mean % area occupied by crops assessed for source and sink effects, uncultivated habitats, and urban development in 3000-m rings surrounding sampled cotton fields. Standard errors are in parentheses. (DOCX) [file pone.0039862.s003.docx]

**Table S1.** Mean % area occupied by crops assessed for source and sink effects, uncultivated habitats, and urban development in 3000-m rings surrounding sampled cotton fields. Standard errors are in parentheses

| Habitat | Year | | |
| --- | --- | --- | --- |
|  | 2007 | 2008 | 2009 |
| Cotton | 30.2 (0.8) | 12.9 (1.3) | 18.9 (1.9) |
| Forage alfalfa | 7.9 (1.4) | 5.5 (1.1) | 8.0 (1.2) |
| Safflower | 1.0 (0.3) | 9.5 (1.4) | 5.3 (1.5) |
| Seed alfalfa | 1.8 (0.4) | 0.3 (0.1) | 0.5 (0.2) |
| Sugar beet | 1.2 (0.4) | 0.3 (0.1) | 0 |
| Tomato | 10.8 (1.4) | 15.6 (1.8) | 11.6 (1.4) |
| Uncultivated habitats | 15.8 (1.6) | 6.6 (0.5) | 8.0 (0.5) |
| Urban development | 1.4 (0.4) | 2.9 (0.8) | 1.7 (0.6) |
